# Development and Evaluation of Monoclonal Antibodies against CBPP Antigen with the End Goal of Developing an ELISA Kit

**DOI:** 10.1155/2024/6901355

**Published:** 2024-05-07

**Authors:** Lorato Ramathudi-Dunbar, Emmanuel Awosanya, Sanne Bodjo Charles, Ethel Chitsungo, Cisse Rahamatou Moustapha Boukary, Nick Nwankpa, Hassen Gelaw, Yebechaye Tessema, Gelagay Melesse A., Richard Rayson Sanga, Adorbley Bright, Jean de Dieu Baziki

**Affiliations:** ^1^Pan African University Life Sciences Institute Including Health and Agriculture (PAULESI), University of Ibadan, Ibadan, Nigeria; ^2^African Union Pan African Veterinary Vaccine Centre (AU-PANVAC), P.O. Box 1746, Debre Zeit, Ethiopia

## Abstract

Contagious bovine pleuropneumonia (CBPP) is an infectious and contagious bacterial respiratory disease that affects cattle with significant economic losses to the African animal industry. The use of ELISA kits based on monoclonal antibodies (mAbs) will aid in quick and precise diagnosis of CBPP, contributing to disease control and prevention in cattle. Thus, this research aims to develop and evaluate monoclonal antibodies against CBPP (T1/44) antigen for use in ELISA kits for CBPP diagnosis. Hybridoma technology was used to develop monoclonal antibodies that recognize and bind to the CBPP (T1/44) antigen. The antibody-secreting hybridomas were produced after immunizing mice with purified CBPP antigens. The hybridomas were screened for high sensitivity, specificity, and liking to the antigen. The selected mAbs were assessed for sensitivity and specificity against CBPP antigen using different immunoassays, dot-blot, ELISA, and mouse mAb isotyping. The monoclonal antibodies were profoundly specific, with a higher hindrance to CBPP antigen (<0.50 OD) while lacking cross-reactivity to other antigens. The monoclonal antibodies could distinguish CBPP antigen at low concentrations, showing their high sensitivity (>80% PI). The isotyped mAbs of intrigued appeared to have a place in the IgG class. These identified monoclonal antibodies can be utilized to develop an ELISA kit for CBPP diagnosis, which would give a fast, precise, and cost-effective strategy for screening and checking CBPP in cattle herds.

## 1. Introduction

Contagious bovine pleuropneumonia (CBPP) is a highly transmissible bacterial disease that principally affects cattle. It is caused by Mycoplasma mycoides subspecies mycoides (Mmm) [1, 2]. It has traits of prolonged cough, fever, and difficulty breathing. Predominantly, the disease is contagious, and it spreads through direct contact with cough droplets, expedited by the crowding of animals [3]. The World Organization for Animal Health (WOAH) listed CBPP among notifiable diseases that are rampant in Africa [4, 5]. The disease was initially isolated in Europe in the early 16th century but later spread to other areas of the world. It has been eradicated in numerous parts of the world but is still persistent in sub-Saharan Africa [3, 6]. This disease can lead to significant economic losses for farmers and the livestock industry and is an obstacle to trade in numerous African countries, hence lessening the value of animals and the financial gain of numerous value chain stakeholders [7]. The incidence of CBPP also poses a relentless terror to disease-free countries and creates costs in terms of the measures essential to assure the mitigation of the disease. Morbidity rates in susceptible herds can reach up to 90%, while the mortality rates can be up to 50% [8]. Despite the hurdles, several efforts have been put in place to successfully regulate and eradicate CBPP throughout Africa, with various degrees of success. These included stamping out policy, movement control, and mass vaccination, which were achieved through the Pan African Control for Epizootics and Joint Project 28 [1, 9]. These initiatives succeeded in containing the disease and ultimately eradicating it in several Southern African nations. Where other control methods are unsuccessful, the remaining option is vaccination, which is still the most practical strategy for CBPP [10]. The potency of the present CBPP vaccine (T1/44 strain) relies on its usage in a properly executed vaccination program that attains a substantial coverage rate of over 80% [11]. It is anticipated that by the first half of the 20th century, CBPP will be eradicated from Africa through vaccination [10]. It is, therefore, crucial to modernize the existing diagnostic techniques to guarantee the availability of the latest and advanced devices. Efficient and reliable detection systems that swiftly identify CBPP antigens are desperately required, especially in Africa where the use of PCR may not always be feasible and high-volume testing is essential [5]. With this, more readily available and affordable techniques, such as ELISA using better antigens, enabling mass screening is necessary [12]. Development of an ELISA test kit for CBPP will provide a better diagnostic kit for CBPP, which is presently expensive and limited after the phase-out of the company, which was producing the kit. The goal of this study was to develop and evaluate monoclonal antibodies against CBPP antigen for ELISA kit development.

## 2. Materials and Methods

### 2.1. Preparation of Mycoplasma Mycoides Subspecies Mycoides (Mmm) Antigen

The CBPP vaccine seed (Mmm, T1/44 strain) obtained from the vaccine seed bank (AU-PANVAC) was utilized for the mycoplasma culture, carried out following the AU-PANVAC CBPP vaccine production method. Concisely, the lyophilized vaccine seed was diluted in 2 ml of pleuropneumonia-like organisms (PPLOs) media (Difco, MD21152, USA) incorporated with horse serum 20% (Sigma-Aldrich, St. Louis, M063103, USA). Then, the mixture was inoculated in 98 ml of PPLO complete media placed in a 200-ml bottle, closed, and incubated with 5% CO_2_, 37°C for three days until the desirable turbidity was observed. The inoculum was then passaged at 1/10 in production media, PPLO-supplemented media in 1000 ml, and harvested after 48 hours. Subsequently, the culture was centrifuged at 12000 rpm through washing three times with PBS. The resuspended antigen was inactivated by adding 0.1% Triton X-100 before mice inoculation.

### 2.2. Generation of Monoclonal Antibodies

The BALB/c mice (*n* = 4, 4 weeks old) were intraperitoneally (IP) immunized with 200 *µ*l of crude CBPP T1/44 antigen emulsion at a concentration of 0.3 mg/ml together with the Freund's complete adjuvant mixed at a 1 : 1 ratio for antibodies production against CBPP antigen, as described by [13, 14]. Booster injections were administered every two weeks. The first three booster injections were composed of the CBPP antigen and incomplete adjuvant mixed at the same ratio as the first injection. The last booster injection (fourth) was 200 *µ*l crude CBPP antigen in saline without adjuvant. Four days after the last boost, the mice were sacrificed, and the spleen cells were prepared (as described by [15]; StemCell Technologies, ClonaCell™-HY Kit, Canada) for fusion with the tumor Ag8 myeloma cells (FAO/IAEA Laboratories, Austria) (prepared as described by [16] and StemCell Technologies, ClonaCell™-HY Kit, Canada) for the production of hybridoma cells. The hybridoma fusion, selection, and harvesting were carried out step by step according to the ClonaCell™-HY Kit (StemCell Technologies, Canada) [15].

### 2.3. Screening of Hybridomas

The supernatant of each hybridoma was analyzed using an indirect ELISA to screen for antibody production against the CBPP antigen, following the protocol of [17] with some modifications. In brief, ELISA plates (NUNC-MaxiSorp, Denmark) were first coated with 100 *µ*l per well of the antigen solution, diluted at 1 : 100 in dilution buffer, and incubated at room temperature overnight. Then, the plates were rinsed thrice with PBS-T composed of Tween 20 (0.05%) and PBS (0.002 M) to eliminate boundless antigens. Using 200 *µ*l/well of blocking solution (5% skimmed milk in PBS-T (Milk-PBS-T)), the unoccupied binding sites in the plate wells were blocked and the plates were incubated at 37°C for 30 minutes. Subsequently, the blocking solution was cast off, and 50 *µ*l of the blocking solution was added in every well followed by 50 *µ*l/well of the medium from hybridoma cell culture (serum containing Abs against CBPP Ag). Then, the plates were incubated for 30 minutes at 37°C and afterwards were washed as delineated above. Next, the anti-mouse immunoglobulin per-oxidase-labeled conjugate (DAKO Company, Denmark) was added (100 *µ*l/well) and diluted at 1 : 100 in PBS-T-Milk, and the plates were further incubated at 37°C for 30 minutes. Then, the plates were rinsed again, followed by the pipetting 50 *µ*l/well of chromogenic substrate 3,3′,5,5′-tetramethylbenzidine (TMB) (Thermo Scientific, Rockford, IL 61101, USA), and incubated for 15 minutes at 37°C until color development was observed. Then, the color development reaction was stopped by adding 50 *µ*l/well of sulfuric acid (1M), and the optical density (OD) of each well was read using a spectrophotometer reader with a 450-nm wavelength. Expanding and subcloning of the hybridomas was performed according to the ClonaCell™-HY Kit (StemCell Technologies, Canada).

### 2.4. Screening for mAbs

Indirect ELISAs (iELISAs) were performed to screen for mAb-positive clones, following the same protocols as described above for the screening of hybridomas following [17] with some modifications.

### 2.5. Mass Production of mAbs

Positive mAb-producing hybridomas cells were grown in serum-free Gibco Hybridoma-SFM (1X) medium ([+] L-glutamine) and were incubated at 5% CO_2_, 37°C. Collection of the medium (cell culture supernatant fluid) was performed once it changed color, indicating that the mAbs have been produced and released into the medium. The fresh medium was added all the time after collection, and further incubation was performed under the same conditions. The collected mAbs were stored at +4°C before precipitation. Some mAbs were cryopreserved following the protocol as described in the cryopreservation of hybridomas.

### 2.6. Purification of mAbs

To generate immunoglobulins (Igs), Mmm mAb-positive hybridoma cells were precipitated and incubated overnight at +4°C in 50% saturated ammonium sulfate (SAS) (Thermo Scientific, Rockford, IL 61101, USA). The Igs were then resuspended in sterile PBS for dialysis to remove the ammonium sulfate, which was used for mAb precipitation. This was performed using the Slide-A-Lyzer dialysis cassettes (Thermo Scientific, Rockford, IL 61105, USA, 2160728) with the membrane molecular weight cutoff (MWCO) filter at the exclusion limit of 10 K, following the kit protocol.

### 2.7. Bicinchoninic Acid Assay (BCA) Protein Quantification, Horseradish Peroxidase (HRP) Conjugation of the CBPP mAbs, and Antibody Isotyping

The total protein quantification of each mAb was performed using the Thermo Scientific™ Pierce™ BCA Protein Assay kit following the manufacturer's protocol and as described by [18] with some changes. Succeeding the quantification of each mAb, the CBPP mAbs were coupled with HRP, shadowing the EZ-Link Plus Activated Peroxidase kit method (Thermo Scientific, Rockford, IL61101, USA). The mouse mAb isotyping was characterized utilizing the Pierce™ Rapid Antibody Isotyping Kit-Mouse (Thermo Scientific, Rockford, IL 26178 or 26179, USA), according to the kit protocol.

### 2.8. Dot-Blot Testing of the mAbs

The dot-blot was performed following the protocol in [19], with some modifications to determine the cross-reactivity or specificity of the mAbs to the CBPP T1/44 antigen relative to other mycoplasmas. The test was performed using all the CBPP (seven) mAbs and one CCPP mAb, and the samples included the CBPP T1/44 antigen, which served as the positive control, CBPP T1sr antigen, CCPP antigen, mycoplasma-contaminated sample (PPR), and PBS (negative control). This was performed using the PVDF membranes (Novex, Lot 2363142, USA). In brief, marks were done on the membranes where the samples were spotted, and the membranes were put in the Petri dishes labeled for each mAb. Then, the antigen samples were spotted on the membranes at the designated spots, and the membranes were incubated nightlong at 37°C to allow the samples to dry. Tailing incubation, the membranes were rinsed with washing buffer (PBS-T) thrice to get rid of the free antigen. Then, the blot membranes were blocked with 5% blocking buffer (PBS-T containing 5% skimmed milk) and were incubated at 37°C for 30 minutes. Subsequently, the milk-PBS-T was thrown away and the membranes were rinsed three times with the washing buffer. Then, the conjugates diluted at 1 in 10 000 in milk-PBS-T were pipetted to the membranes in Petri dishes, ensuring that the membranes were fully submerged in the solution. The membranes were then incubated for an hour under similar conditions as the previous incubation. Following this, the solutions were cast off, membranes were washed as previously, and the TMB was added and then incubated for 15 minutes at 37°C. Then, membranes were observed for color development at the antigen's spots.

### 2.9. Optimization of Reagents for ELISA

#### 2.9.1. Antigen Titration for Selection of Coating Antigen Dilution

The antigen titration was performed to determine the coating antigen dilution for the ELISA plate. This was performed following the protocol in [20], with some modifications. In brief, the CBPP antigen was prepared at a 1 in 50 dilution in PBS-T. Then, in a 96-well ELISA plate, PBS (100 *µ*l) was pipetted to rows 2 to 12 rows. Succeeding that, 100 *µ*l of the CBPP Ag was added to wells in rows 1 and 2. Titration of the antigen by serial dilutions was then performed from wells 2–12. Afterwards, the plate was allowed to incubate night long at ambient temperature. Following incubation, the plate contents were discarded and the plate was washed thrice with washing buffer (PBS-T). Afterwards, 5% milk-PBS-T (200 *µ*l) was pipetted very well and the plates were incubated at 37°C for 30 minutes. Later, the PBS-T-milk was discarded, and consecutively, 100 *µ*l of CBPP serum containing antibodies against CBPP antigen, with known concentration, diluted at 1 : 100 in PBS-T-milk was pipetted to the whole plate. The plate was further incubated at 37°C for 30 minutes. The contents were thrown away, and the plate was rinsed as abovementioned. HRP antibovine conjugate diluted at 1 : 5000 was then pipetted to all the wells (100 *µ*l/well), and the plate was further incubated for an hour under the same conditions. Washing was performed, 50 *µ*l of the substrate solution (TMB) was pipetted across the plate, and the plate was permitted to brood for 15 more minutes. Subsequently, 50 *µ*l of the stop solution (H_2_SO_4_) was added to the whole plate and, immediately, the absorbance was measured using the ELISA plate reader using the 450-nm wavelength filter.

#### 2.9.2. Conjugate Titration

Selection of the conjugates was done by titration of all the conjugated mAbs against the antigen following a similar protocol as for antigen titration, with some changes. In brief, the ELISA 96-well plate was coated with 100 *µ*l per well of CBPP antigen diluted at 1 : 100 and incubated all night at ambient temperature. The following day the plate was rinsed thrice with washing buffer and blocked as above. Then, the plate contents were discarded and 100 *µ*l of blocking buffer was added to wells in columns 2 to 12. Then, 100 *µ*l of each conjugate diluted at 1 : 5 was added to the well in columns 1 and 2, and the serial dilutions were performed from columns 2 to 12. The plate was incubated for an hour under the same conditions as above. Subsequently, the plate was rinsed and then TMB and the stop solution were added followed by the plate reading, as above.

#### 2.9.3. Titration of the Controls

Titration of the CBPP-positive and CBPP-negative controls was carried out to determine the optimum dilution concentrations of the positive and negative controls for maximum antigen-binding inhibition by the positive control. This was performed following the method in [21] with some modifications. Concisely, the 96-well ELISA plate was coated with CBPP antigen diluted at 1 : 400 all night at ambient temperature. Then, the plate was washed thrice and blocked as abovementioned. The plate contents were then discarded, and the blocking buffer was pipetted to the plate wells in columns 2–12. The controls diluted at 1 : 5 were then added to wells in columns 1 and 2; consequently, serial dilutions were performed from columns 2–12, and the plate was further incubated for an hour as previously. After that, the plate was washed three times and the conjugate (selected P3C1) diluted at 1 : 600 was added in all wells, followed by a 1-hour incubation at 37°C. The TMB and the stop solution were added, followed by plate reading, as abovementioned.

### 2.10. Data Analysis

Microsoft Excel version 2311 was used in analyzing the data generated. Descriptive statistics such as frequencies and percentages were calculated. Data were presented in figures and charts.

## 3. Results and Discussion

### 3.1. Results

  3.1.1. Antigen titrations
  3.1.2. Screening mice sera for antibody production
  3.1.3. Screening hybridoma mAbs
  3.1.4. BCA protein quantification of the mAbs
  3.1.5. HRP-conjugated mAb titrations
  3.1.6. HRP-conjugated mAb screening
  3.1.7. CBPP control titration
  3.1.8. Control titrations and percentage inhibition determination
  3.1.9. Titrations of samples using CBPP conjugates to check inhibition
  3.1.10. Percentage Inhibition of samples titrated with HRP-conjugated CBPP mAbs
  3.1.11. Checking cross-reactivity of CBPP conjugates with CCPP sample via titration
  3.1.12. Dot-blot test of the mAbs
  3.1.13. Isotyping the CBPP mAbs

### 3.2. Discussion

A specific and sensitive test for CBPP is necessary for disease surveillance [18]. To precisely detect the CBPP antigen, a specific ELISA test is vital to avoid false detection of other mycoplasmas antigen infections as the CBPP antigen infection [22]. This will hence make the control of CBPP infections easier and will greatly contribute to the disease eradication in the areas where it is still persistent, more especially in some parts of Africa where it heavily affects the farmers' and countries' economies [3].

The development of a sensitive and specific ELISA test kit is necessary to curb the urgent need for the test kit, which is currently unavailable worldwide, to keep up with the disease surveillance. The use of mAbs for the development of ELISA tests for the detection of specific antigens is a promising strategy for precise detection and diagnosis of CBPP, as well as for potency tests in the quality assurance of CBPP vaccines. The developed mAbs have shown promising sensitivity and specificity against the CBPP T1/44 antigen and, hence can be used for the development of an ELISA test kit.

In this study, the aim was to develop and evaluate monoclonal antibodies against CBPP T1/44 antigen for ELISA development, hence seven (7) mAbs were successfully developed. A series of tests were conducted in the process of evaluating the good specific and sensitive mAbs that can be used for the CBPP ELISA kit. A successful propagation of the CBPP T1/44 antigen was developed.

**Figure 1:**
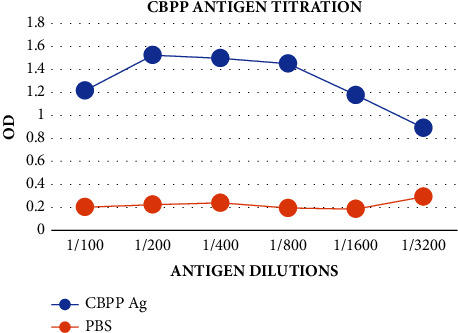
Titration of the CBPP antigen for the selection of the antigen dilution for plate coating as a way of reagent optimization using the 1 : 100 dilution proved the 1 : 400 dilution to be very optimal for ELISA test antigen preparations.

For optimization of reagents for the ELISA tests, the titrations of the antigen to determine the antigen dilution to use for coating the ELISA plate were carried out using the indirect ELISA (iELISA). It was found that the 1 : 100 dilution can be used for antigen dilutions. However, after further titrations using the 1 : 100 dilution in Figure 1, it was found that the optimum dilutions ranged between 1 : 200 and 1 : 800; hence, it was concluded that the 1 : 400 dilution can be used for plate coating for ELISA tests. There was optimum binding of the antigen to the antibodies of known concentration at this dilution.

**Figure 2:**
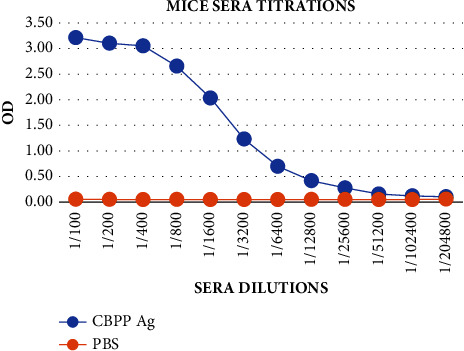
Titration of the blood serum collected from the mice postinoculation with CBPP antigen was screened to determine antibody production against the CBPP antigen. The results demonstrated that antibodies were produced against the CBPP antigen after being subjected to antimouse conjugate (OD > 0.50), at dilutions 1 : 100–1 : 800.

Postimmunization of the mice with CBPP antigen for antibody production, the sera collected were screened using the indirect ELISA to check the production of the antibodies by the inoculated mice. The serum was screened using the HRP-antimouse conjugate with known concentration. The sera showed inhibition of the conjugate binding to the antigen, proving the presence of the antibodies against the CBPP antigen, as shown in Figure 2. The CBPP-positive serum presented OD higher than 0.5, which is the recommended cut-off point by the WOAH [12]. The obtained results are in line with the findings of (20), which showed that sera from CBPP-infected and vaccinated cattle presented OD greater than 0.2, while the sera from noninfected or nonvaccinated cattle presented OD less than 0.2 (0.2 OD was set as a cut-off point) when screened using iELISA.

**Figure 3:**
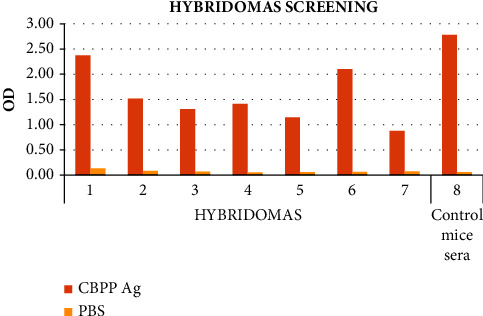
Seven (7) hybridomas identified using iELISA showed to be producing the monoclonal antibodies against the CBPP antigen. The hybridomas were selected based on the OD higher than 0.50.

A total of seven (7) hybridomas showed to be producing the antibodies against CBPP antigen and were selected for expansion processes following the iELISA screening. The hybridomas were selected based on an OD higher than 0.50, which indicated higher binding of the antibodies to the CBPP antigen, as portrayed in Figure 3. This agrees with the WOAH [12], which stated that the cutoff point for testing the mAbs is 50% for the ELISA test. This proved that their hybridomas were producing monoclonal antibodies (mAbs) against the CBPP antigen and could be subjected to further investigations. Studies carried out by [23] showed that clones screened for relative strength of antigenicity demonstrated reactivity of more than 30 OD % concerning the control.

**Figure 4:**
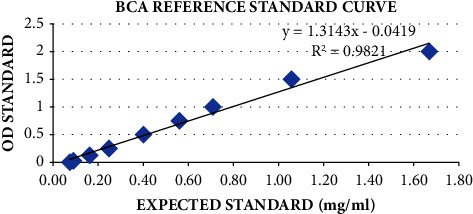
The standard curve for the BCA protein quantification of the mAbs. This was utilized to determine the protein quantity of the mAbs.

The BCA protein quantification of the mAbs was conducted, and the *R*^2^ value of 0.98 was obtained, as depicted in Figure 4. This proved a positive correlation between the optical density obtained and the amount of protein as in [13]. The standard curve was utilized as a reference to determine the protein quantity obtained (in mg/ml) of the CBPP mAb samples. All seven mAbs showed a protein concentration greater than 1.50 mg/ml. The obtained concentrations were utilized for HRP conjugation of the mAbs.

**Figure 5:**
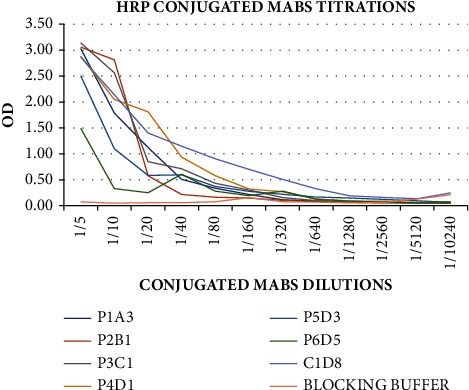
Titrations of all the HRP-conjugated mAbs against the CBPP antigen were carried out for optimization of the conjugate dilutions to be used for the ELISA tests. At 1 : 5 dilution, conjugates demonstrated greater binding.

**Figure 6:**
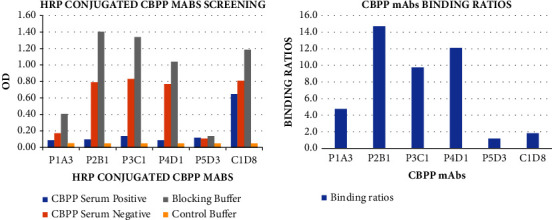
Screening of mAbs and their binding ratios relative to the CBPP strong positive serum. The mAbs P2B1, P3C1, and P4D1 showed higher OD (>0.50) and binding ratios (≥10), hence qualifying them to be selected for use in further ELISA investigations.

All the mAbs conjugates were subjected to titration after HRP conjugation to determine their optimum conjugate dilution ratio for setting up the ELISA test. The conjugates showed greater binding at the 1 : 5 dilution, as shown in Figure 5; therefore, the dilution was used for the subsequent tests. The HRP-conjugated CBPP mAbs were screened, and the obtained OD, as shown in Figure 6 (top), was used to calculate the binding ratios, Figure 6 (lower). The mAbs P2B1, P3C1, P4D1, and C1D8 showed OD higher than 0.50 relative to the negative serum and the blocking buffer. The OD of the CBPP strong positive serum relative to the blocking buffer was utilized to determine the binding ratios of the mAbs. The binding ratios represent the number of antibodies bound to the antigen [13]. The mAbs with high binding ratios (>50) were selected to use for further investigations. Therefore, conjugates P2B1, P3C1, and P4D1, which presented higher OD and BR, were selected for use in further investigations.

**Figure 7:**
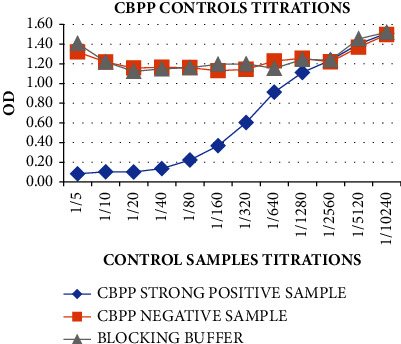
Titrations of the CBPP strong positive and negative controls against CBPP mAbs to determine the optimum dilution for the controls to be used for the ELISA setup. The CBPP strong positive control showed greater binding inhibition of the conjugates from 1 : 5 to 1 : 640 dilutions.

The HRP-labeled mAbs were used for the titrations of the CBPP strong positive and negative controls against CBPP mAbs to determine the optimum dilution for the control to be used for the ELISA setup. The curve in Figure 7 displayed that the dilutions 1 : 5–1 : 20 can be utilized for the controls as there was high mAbs-binding inhibition (>50) by the CBPP strong positive control sample collated with the CBPP-negative control and the control buffer. Therefore, the 1 : 10 (median) concentrations of the controls were then found to be the optimum for use in the ELISA test. The CBPP-negative control sample proved to be a good candidate for the negative control as it gave almost the approximate OD as the control buffer proving no inhibition of the mAbs against the CBPP antigen.

**Figure 8:**
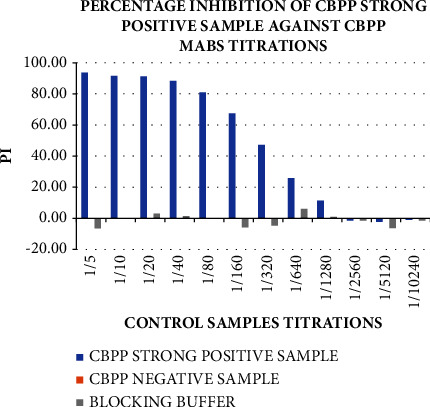
The strong positive and negative control samples' percentage inhibition was determined using the control titration results in Figure 7. The CBPP strong positive control showed greater inhibition of the mAbs binding relative to the CBPP-negative control.

The OD from the control titrations was used to determine the percentage inhibition (PI) of the controls, as shown in Figure 8, to select the positive and negative control for ELISA tests. CBPP strong positive control sample greatly inhibited the binding of the mAbs at 93.75 and 91.23 PI relative to the CBPP-negative control sample. This was in agreement with the WOAH [12, 24] studies, which stated that the cutoff for inhibition is 50%, hence the controls met the set standards. This showed that the CBPP strong positive control sample had the same epitope as the mAbs and hence inhibited their binding to the antigen. This proves our mAbs to be good for the ELISA test in which the positive samples inhibit the binding of the conjugated mAbs from binding to the antigen of interest.

**Figure 9:**
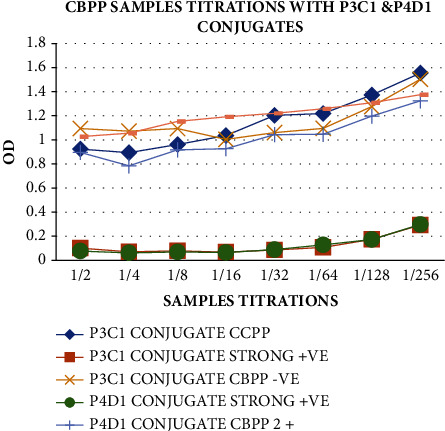
Sample titrations using the selected CBPP conjugates, P3C1 and P4D1, to check the inhibition of CBPP conjugates binding by the CBPP strong positive sample, which proved greater inhibition (OD < 0.50) (P2B1 and P3C1 always showed similar results; hence, for this test, P3C1 was selected to represent the duo).

Titration of CBPP-positive and CBPP-negative serum samples and CCPP-positive serum samples using P3C1 and P4D1 conjugates to determine the binding inhibition of the conjugates. The results in Figure 9 show a great inhibition of both conjugates by the CBPP strong positive sample with OD < 0.50, which had a lower OD compared to other samples. This shows that the CBPP is strongly positively bound to the CBPP antigen, thus inhibiting the binding of the conjugated mAbs, which are specific to the CBPP antigen.

**Figure 10:**
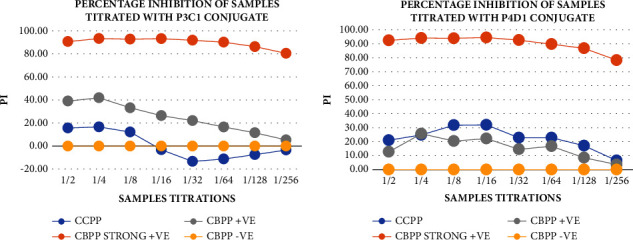
The percentage inhibitions of the sample titrations relative to the negative control using the mAb conjugates P3C1 and P4D1. The CBPP strong positive control showed high sensitivity by extremely inhibiting the binding of the mAbs with more than 80% PI.

Based on the titration OD, the percentage inhibitions of the samples were calculated and the CBPP strong positive control sample was shown to be extremely inhibiting the binding of the mAbs with more than 80% PI, as depicted in Figure 10. The PI of the other CBPP sample was also higher than that of the CCPP and control negative CBPP sample, which presented a PI of less than 40%. However, with the P4D1, the CCPP sample presented a PI little higher than that of the CBPP-positive sample. The obtained results relate to the findings of [25], which showed that ELISA-tested cattle serum samples presented PI of 50% or more for positive sera, between 40 and 50% for doubtful and less than 40% for negative sera. This proves that the mAbs have high sensitivity in detecting the CBPP-positive samples. This is a trait of the good mAbs, which enables them to be capable of distinguishing between the positive and negative samples. This correlates with the findings of [21], which showed that the CBPP-positive screened with iELISA have a high sensitivity of more than 80%.

**Figure 11:**
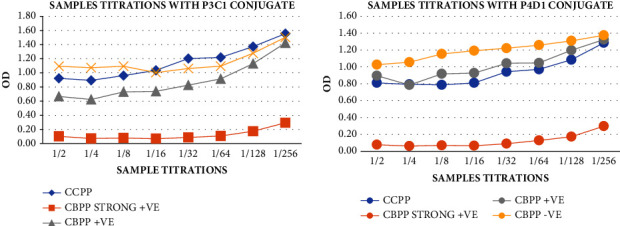
Titrations of the samples against the mAbs to check the epitope cross-reactivity of the CBPP mAb conjugates with the CCPP samples. All the conjugates powerfully reacted with the CBPP strong positive control, showing that they have the same epitope, hence greater binding inhibition of the mAb conjugates.

Cross-reactivity of the CBPP conjugates with the CCPP was determined by titrations of the samples against the mAbs, as presented in Figure 11. Conjugate P3C1 was selected with the P4D1 for this test to check for the epitope cross-reaction of the two mAbs (if they have different epitopes). All the mAbs conjugates were shown to be strongly reacting with the CBPP strong positive control sample, showing that they have the same epitope, hence greater binding inhibition of the mAb conjugates, followed by the CBPP-positive sample, while there was less reactivity with the CCPP and the negative samples. This shows that the mAbs have stronger specificity and sensitivity toward the CBPP-positive samples than the negative, hence will make a good test for detecting the CBPP-infected sera.

**Figure 12:**
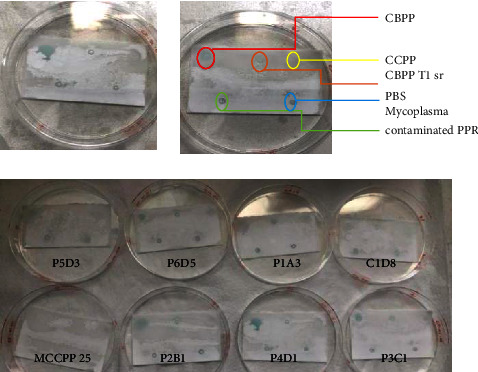
The dot-blot of all the mAbs showed color development only on the CBPP T1/44 sample as compared to other samples, proving specificity and no cross-reactivity with other mycoplasmas.

The dot-blot test was performed on samples using all the mAbs and the mAbs to confirm the cross-reactivity of the CBPP mAbs with other mycoplasmas. As shown in Figure 12, the mAbs did not show to be cross-reacting with other mycoplasma antigens, such as CBPP T1sr, CCPP, and mycoplasma-contaminated (PPR) samples. The color development was visible on the CBPP T1/44 antigen sample, while there was no color development on the other samples as well as the control buffer (PBS). Therefore, the mAbs were shown to be specific to the CBPP T1/44 antigen. This proves the mAbs to be good since they will not falsely detect any other mycoplasmas except the CBPP T1/44 antigen. To prove specificity, the HRP-conjugated mAbs were expected to react to the CBPP T1/44 antigen that they are developed against and produce color upon addition of the TMB substrate. In comparison, the negative PBS did not react to the mAbs, and hence, there was no color development, which agrees with [19]. According to [22], this method is sensitive and trustworthy for verification of CBPP infection.

**Figure 13:**
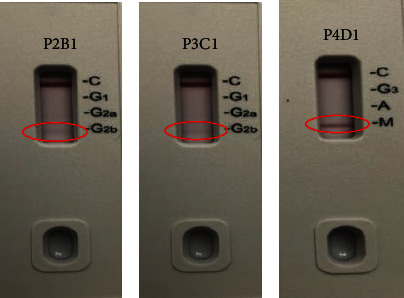
The isotyping test of the selected CBPP mAbs. The figure (from left to right) shows the results' representation of mAb P2B1 and P3C1 (IgG2b) and mAb P4D1 (IgM).

The selected CBPP mAbs were subjected to isotyping to determine their monoclonal class and subclass identity. The selected mAbs P2B1 and P3C1 showed to belong to the IgG class, subclass 2b, while the P4D1 belongs to the IgM class, as presented in Figure 13. Based on the acquired results, the mAbs P2B1 and P3C1, which belong to class IgG, can be used for ELISA test and kit development, while the P4D1 can be used for the rapid immunocapture for immediate infection tests. The results are colligating with the findings of [26], which state that the plasma of cattle infected with CBPP express significant levels of IgG2 or IgM but exhibit low levels of T cells, which results in suppression of the expression of IgA and IgG1 in the plasma.

## 4. Conclusion and Recommendations

### 4.1. Conclusion

The specificity and sensitivity of the developed and evaluated CBPP mAbs show that two of them (P2B1 and P3C1) have the potential to be utilized for the development of an ELISA test against CBPP antigen, while one mAb demonstrated that it can be utilized for the development of a rapid immunocapture test for immediate infection. Other mAbs can be utilized for other research purposes.

### 4.2. Recommendations

It can be recommended that, for further studies, there is a need to test more positive samples from the field (CBPP-infected samples) and negative samples from CBPP-free countries to validate and optimize the obtained results as well as to determine the cutoff points, which requires lots of positive and negative samples.

## Data Availability

The data used in this study can be made available upon valid request.
